# Neurology

**Published:** 1899-10

**Authors:** William C. Krauss

**Affiliations:** Buffalo, N.Y.


### NEUROLOGY.

Conducted by WILLIAM C. KRAUSS, M. D., Buffalo, N. Y.

### CLASSIFICATION OF MENTAL DISEASES.

STUDENTS of mental disease (*Med. Age*) will be interested in the system of classification propounded by Dr. Andriezen, based, as he considers, upon fundamental facts of evolution. There are five main groups of insanity, and the words used to designate them explain themselves: (1) aphrenia, arrest of cerebral development with absence or deficiency of evolution in personality; (2) oligophrenia, enfeeblement of cerebral development with a parallel enfeeblement in the evolution of personality; (3) paraphrenia, anomalies and perversions of cerebral development with corresponding evolution of personality; (4) phrenopathia, morbid conditions or derangements occurring in brains of nearly full development with previous apparent health, with corresponding morbid alterations of the personality; and (5) lipophrenia, terminal conditions of mental dissolution secondary to previous insanities. The insanities thus studied fall into five groups which assume serial arrangement.

---

### ACROMEGALY.

DRS. MITCHELL and Le Count (*New York Medical Journal*, April 29, 1899,) from their study of the pathology of this affection, are led to the following conclusions:

1. The cases of acromegaly associated with true tumor of the hypophysis are certainly not as numerous as has been heretofore supposed.

2. There is not as much constancy in the pathologic condition of the hypophysis as there is in an enlargement of the heart, the thyroid gland, or the sella turcica.

3. Acromegaly does not depend, at least not solely, upon abolition of any function of the hypophysis.

4. A relationship between the thyroid gland and the hypophysis has already been amply proved.

5. It is not at all improbable that proliferation of the histological elements of the hypophysis may be instituted in some cases by a primary enlargement of the sella turcica; in other cases, an edema or hemorrhage *in vacuo*.

6. There is no reason for supposing that enlargement of the sella turcica may not be as constant an occurrence in acromegaly as the changes in other bones, or that it might not take place from a similar cause or causes.

---

### EXPERIMENTAL STUDY OF CHILDREN, MORE ESPECIALLY OF WASHINGTON SCHOOL CHILDREN.

MR. ARTHUR MACDONALD (*St. Louis Medical and Surgical Journal*, July, 1899,) the specialist in the Bureau of education in Washington, studied 1,074 school children with regard to several lines of investigations as follows :

1. Cephalic index and locality upon the skin, with relation to sex, mental ability and sociological conditions.

2. An anthropometrical and sociological study of school children, based upon measurements by the teachers.

3. A purely psychological inquiry as to comparative mental ability in the different school studies as reported by the teachers.

4. A study of the abnormal children.

Conclusions as to 1,074 children specially studied :

1. Dolichocephaly, or long-headedness, increases in children as ability decreases. A high percentage of dolichocephaly seems to be a concomitant of mental dulness.

2. Children are more sensitive to locality and heat on the skin before puberty than after.

3. Boys are less sensitive to locality and more sensitive to heat than girls.

4. Children of the nonlaboring classes are more sensitive to locality and heat than children of the laboring classes.

5. Colored children are much more sensitive to heat than white children. This probably means that their power of discrimination is much better, and not that they suffer more from heat.

Conclusions as to all the school children :

6. As circumference of head increases mental ability increases.[1]

7. Children of the nonlaboring classes have a larger circumference of head than children of the laboring classes.

8. The head circumference of boys is larger than that of girls, but in colored children the girls slightly excel the boys in circumference of head.

9. Colored girls have larger circumference of head at all ages than white girls.

---

[1] It being understood that the race is the same.

10. An important fact already discovered by others is that for a certain period of time before and after puberty, girls are taller and heavier than boys, but at no other time.

11. White children not only have a greater standing height than colored children, but their sitting height is still greater; yet colored children have a greater weight than white children—that is, white children, relatively to their height, are longer bodied than colored children.

12. Bright boys are in general taller and heavier than dull boys. This confirms the results of Porter.

13. While the bright colored boys excel the dull colored boys in height, the dull excel the bright in sitting height. This seems to indicate a relation or concomitancy of dulness and long-bodiedness for colored boys.

11. The pubertal period of superiority of girls in height, sitting height, and weight is nearly a year longer in the laboring classes than in the nonlaboring classes.

15. Children of the nonlaboring classes have, in general, greater height, sitting height, and weight than children of the laboring classes. This confirms the results of investigations by Roberts, Baxter and Bowditch.

16. Girls are superior to boys in their studies (but see conclusion 19).

17. Children of the nonlaboring classes show greater ability in their studies than children of the laboring classes. This confirms the results of others.

18. Mixture of nationalities seems to be unfavorable to the development of mental ability.

19. Girls show higher percentages of average ability in their studies than boys, and therefore less variability. This is interpreted by some to be a defect from an evolutionary point of view, but see conclusion 16.

20. As age increases brightness decreases in most studies, but dulness increases except in drawing, manual labor and penmanship; that is, in the more mechanical studies.

21. In colored children brightness increases with age, the reverse of what is tiue in white children.

Conclusions as to children with abnormalities :

22. Boys of the nonlaboring classes show a much higher percentage of sickness than boys of the laboring class.

23. Defects of speech are much more frequent in boys than in girls.

24. Boys show a much greater percentage of unruliness and laziness than girls.

25. The dull boys have the highest per cent. of unruliness.

26. Abnormalities in children are most frequent at dentition and puberty.

27. Children with abnormalities are inferior in height, sitting height, weight and circumference of head to children in general.

---

### THE RELATION OF HEADACHE TO AFFECTIONS OF THE EYE.

Dr. Samuel D. Risley, of Philadelphia, read a very interesting paper (*Virginia Medical Semi-Monthly*, August 25, 1899,) on headache and eye affections before the neurological section of the American Medical Association, at the Columbus meeting in June, 1899. The author stated that in the last 1,000 eye private patients applying for treatment for all causes, 50 per cent. of them complained of this symptom. A large percentage of these came by the advice of their family physician, or through the persuasion of a friend, complaining of headache alone, and were unconscious of any ocular trouble.

The author pointed out the relation between abnormalities in the shape of the eye-ball upon which errors of refraction ordinarily depend, and the faulty form of the bony orbit associated with certain distortions of the skull, and called attention to this fact as one of the instances in which disease and impairment of function depend upon a congenital anatomical deformity.

The sudden onset of headache during or after middle life, or following recovery from acute disease, does not exclude the eyes as an etiological factor. The main points of the paper were enforced by brief histories of illustrative cases representing different groups of patients.

The paper was then summarised in the following conclusions as established by clinical experience :

1. That abnormalities of the ocular apparatus are, in a large group of patients, the sole and sufficient cause of headache.

2. That these abnormalitiesof vision may be the unsuspected cause and, therefore, the absence of symptoms obviously referable to the eyes does not exclude them as an etiologic factor in headache, insomnia, vertigo, petit chorea in children, and certain stomach derangements.

3. That the recent or sudden development of symptoms—*e. g.*, after attacks of severe illness, as typhoid fever, the exanthemata, or in association with more or less acute exacerbations of some general dyscrasia—is not sufficint evidence against ocular participation in causing the symptoms.

4. That the participation of the eyes as an etiological factor in headache can be positively excluded only in the absence of ocular disease or after the most painstaking correction of any existing error of refraction or abnormality of binocular balance.

5. That, for the relief of reflex symptoms, accurate corrections are essential, and these can be secured only by the more or less prolonged use of a strong cyclophlegic.

6. That immediate relief by these corrections, in a large group of patients, is not to be expected, since the pain is frequently due to associated pathological conditions of the fundus oculi, and these require time for cure.

---

### KERNIG'S SIGN.

In delivering (*Boston Med. and Surg. Jour.*) the Cavendish lecture before the West London Medico-Chirurgical Society on Cerebro-spinal fever, Dr. William Osler spoke as follows on Kernig's sign.

Described by a Russian physician, and studied in Germany and France, this interesting sign has not attracted the special attention of English and American physicians, though J. B. Herrick, of Chicago, at the last meeting of the Association of American Physicians, spoke of its value. It has been present in all of our cases in which it has been looked for. It is, I think, an old observation that the subjects of protracted meningitis, particularly children, very often lie with the thighs flexed upon the abdomen, and with the legs in a state of partial contracture, so that they are with difficulty extended. To test for Kernig's sign the patient should be propped up in bed in the sitting position, then, on attempting to extend the leg on the thigh there is contraction of flexors which prevents the full straightening of the leg. On the other hand, in the recumbent posture the leg can be fully extended. Many patients with meningitis are not in a condition to sit up, and the test can be equally well made by flexing the thigh on the abdomen, when on attempting to extend the leg, if meningitis be present, the limb cannot be fully extended. Friis found the sign in fifty-three or sixty cases, and Netter in forty-five or fifty. It is stated to be present in all forms of meningitis when the spinal meninges are involved. The presence of the sign is no indication of the intensity of the spinal involvement, as it existed in a very marked degree in a recent case of pneumococcic meningitis, in which there was no positive exudate on the spinal meninges, only a turbid fluid. Netter's explanation of the phenomenon is as follows: "In consequence of the inflammation of the meninges the roots of the nerves become irritable and the flexion of the thighs upon the pelvis, when the patient is in a sitting position, elongates and consequently stretches the lumbar and sacral roots, and thus increases their irritability. The attempt to extend the knee is insufficient to provoke a reflex contraction of the flexors while the patient lies on his back with the thighs extended upon the pelvis, but it does so when he assumes a sitting posture.

---

PHYSICAL CONDITION OF THE INSANE.

DR. R. GANTER (*Allgemeine Zeitschrift f. Psychiatrie*) recently studied the physical anomalies of 345 cases of insanity at the asylum at Trier, noting carefully the anomalies of the skeleton, skin, reflexes and sensory organs. The different groups were divided as follows: 119 cases of paranoia, 133 of dementia, 3 of dementia senilis, 12 of paresis, 48 imbeciles and idiots and 30 epileptics. 17.7 per cent. offered no anomalies; 31 per cent. showed 1; 25.5 per cent., 2; 13.9 per cent, 3; 8.1 per cent., 4; 2.9 per cent., 5 and the same number showed 6, 7 or 9 anomalies.

Othematoma were found on sixteen ears among twelve patients, none of whom were paretics.

